# A Multimodal Exertional Test for concussion: a pilot study in healthy athletes

**DOI:** 10.3389/fneur.2024.1390016

**Published:** 2024-04-18

**Authors:** Kyla L. Pyndiura, Alex P. Di Battista, Doug Richards, Nick Reed, David W. Lawrence, Michael G. Hutchison

**Affiliations:** ^1^Centre for Sport-Related Concussion Research, Innovation, and Knowledge, University of Toronto, Toronto, ON, Canada; ^2^Faculty of Kinesiology and Physical Education, University of Toronto, Toronto, ON, Canada; ^3^Defence Research and Development Canada, Toronto Research Centre, Toronto, ON, Canada; ^4^Department of Occupational Science and Occupational Therapy, University of Toronto, Toronto, ON, Canada; ^5^Rehabilitation Sciences Institute, University of Toronto, Toronto, ON, Canada; ^6^Department of Family and Community Medicine, Faculty of Medicine, University of Toronto, Toronto, ON, Canada; ^7^Mount Sinai Hospital, Sinai Health System, Toronto, ON, Canada; ^8^Keenan Research Centre for Biomedical Science, St. Michael’s Hospital, Toronto, ON, Canada

**Keywords:** sport-related concussion, rehabilitation, mild traumatic brain injury, recovery, assessment

## Abstract

**Introduction:**

Exertional tests have become a promising tool to assist clinicians in the management of concussions, however require expensive equipment, extensive spaces, and specialized clinician expertise. As such, we developed a test with minimal resource requirements encompassing key elements of sport and physical activity. The purpose of this study was to pilot test the Multimodal Exertional Test (MET) protocol in a sample of healthy interuniversity athletes.

**Methods:**

The MET comprises four stages, each featuring three distinct tasks. The test begins with engaging in squats, alternating reverse lunges, and hip hinges (*Stage 1*). The next stage progressively evolves into executing these tasks within specified time limits (*Stage 2*). Following this, the test advances to a stage that incorporates cognitive tasks (*Stage 3*), and the final stage demands greater levels of physical exertion, cognition, and multi-directional movements (*Stage 4*). Heart rate (HR) was obtained during each stage of the MET and participants’ symptom severity scores were recorded following each task.

**Results:**

Fourteen healthy interuniversity athletes (*n* = 8 female, *n* = 6 male) participated in the study. HR was obtained for 10 of the 14 athletes (females: *n* = 6, males: *n* = 4). Increases in average and maximum HR were identified between pre-MET and Stage 1, and between Stages 3 and 4. Consistent with the tasks in each stage, there were no increases in average and maximum HR observed between MET Stages 1 to 3. Female athletes exhibited higher average and maximum HRs compared to male athletes during all four stages. All 14 athletes reported minimal changes in symptom severity following each task.

**Conclusion:**

Among healthy athletes, the MET elicits an increase in average and maximum HR throughout the protocol without symptom provocation. Female athletes exhibit higher HRs during all four stages in comparison to male athletes.

## Introduction

The clinical assessment of concussion and determination of recovery has undergone remarkable enhancements over the past 30 years (1–6). While post-concussion symptom evaluation continues to be a crucial component (1), clinical evaluation of concussion has evolved to include a variety of tests that now embrace static and dynamic balance, cognitive functioning, oculomotor performance, vestibular functioning, and dual-task proficiency (7, 8). Furthermore, the implementation of a graduated return-to-sport (RTS) strategy has become an essential component of concussion management (1). This strategy involves a graded escalation in physical exertion; the progression through which requires the absence of symptom exacerbation, in accordance with the 2023 Consensus Statement on Concussion in Sport (1).

One significant limitation of our current understanding of RTS following a concussion is the need for universally accepted thresholds for intensity (i.e., heart rate [HR]), type and complexity of movement, and duration of activity required within each stage of the RTS strategy. Presently, providers and patients primarily rely on the subjective response to exertional stressors to guide progression through the various stages. Healthcare professionals must also make decisions based on their patients’ recall during each step of the RTS strategy in order to inform further RTS recommendations.

Clinical tests have been developed to mitigate these limitations, purporting to offer a more comprehensive and objective evaluation with consideration of athletic-specific contexts. These tests necessitate the concurrent execution of motor (i.e., physical) tasks with visual or cognitive tasks. These multifaceted “dual-task” testing paradigms have demonstrated a capacity to identify performance deficits potentially overlooked by conventional single-domain clinical measures (9, 10). However, it is essential to underscore that the psychometric properties (e.g., reference norms, test-retest reliability) of these dual-task assessments, as well as the standardization of testing procedures across divergent age groups and cultural norms, have not yet been definitively established (11, 12).

While dual-task tests offer a more accurate approximation of the movements and complexities inherent in sport, there are still limitations as the motor elements employed in such tests typically involve a gait or balance task conducted within a single plane of movement, whereby sports often necessitate multi-planar movements, rotations, and accelerations. Both the Gapski-Goodman Test (GGT) (13) and the Dynamic Exertion Test (EXiT) (14), have bridged this gap by amalgamating sensory, motor, and cognitive components. Both of these tests involve an aerobic exercise component and a plyometric/dynamic circuit protocol (13, 14). When these tests are incorporated within the post-concussion medical clearance assessment, they have demonstrated efficacy in identifying a subset of individuals prone to symptom exacerbation (13, 14). Notably, Marshall et al. (13) found that 14.6% of participants experienced symptom provocation during the GGT or the modified GGT, while Kochick et al. (14) observed that 6.6% of patients exhibited symptom provocation on EXiT.

Despite the significant strides made in concussion management from the utilization of dual-task tests, resource constraints such as the reliance on expensive equipment (i.e., treadmill or stationary bike), extensive spaces, and specialized clinician expertise limit their generalizability. To help address these potential barriers, our objective was to develop a test with minimal resource requirements encompassing key essential elements of sport/physical activity, including HR elevation, multi-planar movement, and multi-tasking; all executable within a limited space such as a doctor’s office. The ultimate goal is to develop and validate a generalizable, accessible, user-friendly, and multimodal physical exertion test that captures the key elements of sport participation. By accurately reflecting the multifaceted demands of sport participation, this test is designed to serve as a robust tool in aiding practitioners in the decision-making process for medical clearance, thereby facilitating a safe and informed return to sport for athletes post-concussion. To that end, it is imperative to first ascertain the response of healthy athletes to the Multimodal Exertional Test (MET) to ensure that the test can effectively elicit increases in HR while maintaining symptom provocation to a minimum. Therefore, the purpose of this study was to assess HR responses and associated symptoms at each stage of the MET protocol within a group of healthy interuniversity athletes, thereby establishing a foundational understanding of physiological and symptom responses during exertion.

## Materials and methods

### Study design

The current pilot study evaluated a newly developed Multimodal Exertional Test (MET) among a sample of healthy interuniversity athletes. The study was completed at an academic institution with all participants providing written informed consent prior to enrollment. All study procedures were in accordance with the Declaration of Helsinki and approved by the Health Sciences Research Ethics Board, University of Toronto (protocol #41884).

### Participants

Athletes were recruited during the period from March 2022 through April 2022. Fourteen participants (female, *n* = 8; male, *n* = 6) from seven sports with an average age of 20.0 years old participated in the study. Exclusion criteria for participants included a history of concussion within 6 months of the study assessment and any injuries that would limit the participant from properly performing exercises and/or physical movements, both of which were self-reported. The demographics of the study population are described in the *Results* section.

### Measures

Prior to beginning the MET, participants completed the Hopkins Verbal Learning Test (HVLT) (15, 16), a symptom evaluation, and were fitted with a HR monitor. With all prior components included (i.e., HVLT, symptom evaluation, and applying HR monitor to the body), the MET protocol takes approximately 20–25 min to administer and complete.

#### Multimodal Exertional Test

**Figure 1:**
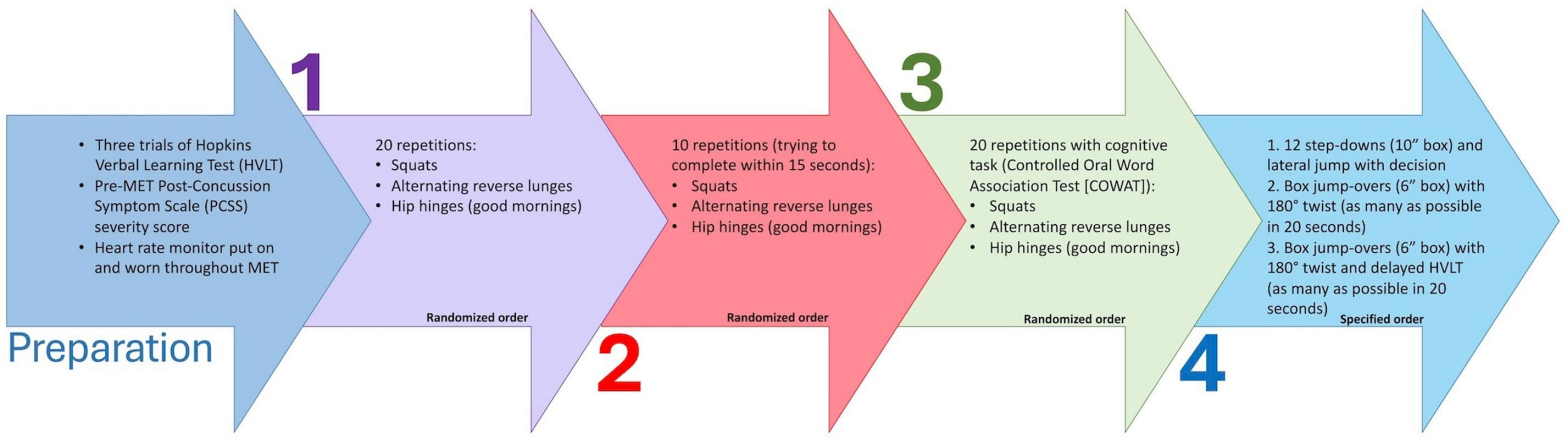
Descriptions of preparation prior to and four stages of the Multimodal Exertional Test (MET) protocol. MET, Multimodal Exertional Test; HVLT, Hopkins Verbal Learning Test; PCSS, Post-Concussion Symptom Scale; COWAT, Controlled Oral Word Association Task. Each stage comprises of three tasks in which the order of tasks is randomized for Stages 1–3, however kept in specified order for Stage 4.

The development of the MET followed De Vet et al. (17) six-step framework for developing a measurement instrument; details of the process and steps can be found in Supplementary Methods 1. Briefly, the MET consists of a four-stage test with three tasks per stage. The MET progressively increases in difficulty by adding a new component at each stage: (Stage 1) cardiovascular load, (Stage 2) head acceleration, (Stage 3) cognitive tasks (i.e., dual-tasks), and (Stage 4) elements of coordination and multi-plane movements (the full protocol can be found in Figure 1).

#### Hopkins Verbal Learning Test

Three trials of the HVLT (15, 16) were completed before beginning the MET. The HVLT is a 12-word memory test where an examiner reads 12 words at a rate of one word per second and the participant is asked to recite as many words as they can remember. This is completed across three trials with the total number of words calculated (maximum score = 36 words). The delayed recall component was completed during the last task of the MET Stage 4, where participants recited the 12 words from memory (maximum score = 12).

#### Symptom evaluation

Participants completed a 27-item Post-Concussion Symptom Scale with each symptom ranked on a 7-point Likert scale from none to severe (0 indicated “none,” 1–2 “mild,” 3–4 “moderate,” and 5–6 “severe”). This symptom evaluation comprised of the 22-item SCAT-5 (18) symptom evaluation with an additional five symptoms from the C3Logix platform (19, 20), including “sleeping more than usual,” “sleeping less than usual,” “difficulty sleeping soundly,” “ringing in the ears,” and “numbness and tingling.” The overall symptom severity score was calculated by summing all rated symptoms (maximum score = 162). Following each task of the MET (12 total tasks), participants were asked if there were any changes to their symptoms and a total symptom severity score was recorded.

#### Heart rate

Participants placed a Firstbeat chest strap HR monitor (Firstbeat Technologies Oy, Jyvaskyla, Finland) upon arrival and wore it for the duration of the MET. Average and maximum HR were calculated before beginning the MET and during each of the four MET stages.

### Statistical analyses

Descriptive statistics were performed for participant demographics. To estimate HR and symptoms across the four stages of the MET, we employed multilevel Student-t models. The modelling was based on the heuristic causal assumption that the MET would elicit an increase in HR without provoking symptoms, and that the estimates of HR and symptoms would differ in males and females. Posterior contrasts were created to estimate the differences across stages and between males and females. Posterior distributions for all estimates were derived using Hamiltonian Monte Carlo as implemented in Stan through RStan (21, 22) (version 2.21) via R (23, 24) (version 4.3). The R packages “rethinking” (25, 26) and “loo” (27) were used to aid in the processing of posterior samples. All models were assessed for convergence by inspection of trace plots, R-hat values, and effective sample sizes. Priors were selected via prior predictive simulation to span a scientifically credible range of outcomes, thus regularizing posterior parameter estimates. For more modeling information, including mathematical notation, prior selection, and posterior predictive checks, please see Supplementary Methods 2 and Supplementary Figures 1–3 and the GitHub repository containing the code accompanying the manuscript.^1^ All plots were created using the R packages “ggplot2” (28), “bayesplot” (29), and “tidybayes” (30) and all tables were created using the “gt” (31) and “gtsummary” (32) packages.

## Results

### Demographics

Participant demographics are reported in Table 1. Briefly, the median age of male (median = 19.1, interquartile range [IQR] = 19.3–20.4) and female (median = 20.1, IQR = 19.3–20.7) participants were similar. Males were on average taller and heavier than females. Males and females also shared a similar concussion history: the majority (males = 62%, females = 67%) reported no prior concussions. Every athlete in the study with a history of concussion had received medical clearance following their most recent incident and was actively engaged in their sport at full capacity. Raw values of MET performance metrics across all four stages can be seen in Table 2.

**Table 1:** Participant demographics.

Characteristic	Female, *N* = 8	Male, *N* = 6
**Demographics**		
Age	20.08 (19.32–20.68)	19.94 (19.10–20.38)
Height (cm)	164 (162–171)	181 (179–186)
Weight (kg)	65 (63–68)	81 (79–98)
**Education Level**		
Undergraduate (Year 1)	4 (50%)	2 (33%)
Undergraduate (Year 2+)	4 (50%)	4 (67%)
**Sport**		
Basketball	1 (13%)	0 (0%)
Field Hockey	2 (25%)	0 (0%)
Football	0 (0%)	3 (50%)
Lacrosse	1 (13%)	1 (17%)
Soccer	3 (38%)	1 (17%)
Track and Field	0 (0%)	1 (17%)
Volleyball	1 (13%)	0 (0%)
Learning Disability	1 (13%)	0 (0%)
Anxiety	2 (25%)	0 (0%)
Depression	2 (25%)	0 (0%)
Headaches/Migraines	1 (13%)	1 (17%)
Number of Prior Concussions		
0	5 (63%)	4 (67%)
1	1 (13%)	2 (33%)
2	2 (25%)	0 (0%)

Data presented as Median (IQR), or *n* (%).

**Table 2:** Raw values of MET performance metrics.

Characteristic	Overall, *N* = 14	Female, *N* = 8	Male, *N* = 6
**Pre-MET**			
HVLT (sum of three trials /36)	27 (24–28)	27 (24–28)	27 (25–28)
**MET—Stage 1**			
20 Hip Hinges (completion time [seconds])	41.6 (36.7–52.5)	48.2 (36.7–56.1)	38.4 (36.7–47.0)
20 Lunges (completion time [seconds])	41.8 (36.5–48.5)	44.8 (37.6–49.7)	40.0 (36.6–41.9)
20 Squats (completion time [seconds])	31.5 (30.5–44.4)	37.1 (30.7–44.4)	31.5 (30.6–41.3)
**MET—Stage 2**			
10 Hip Hinges (completion time [seconds])	14.7 (13.7–16.2)	15.1 (13.5–16.7)	14.4 (13.8–14.9)
10 Lunges (completion time [seconds])	14.5 (13.7–15.4)	14.9 (14.0–16.4)	14.5 (13.6–14.7)
10 Squats (completion time [seconds])	12.8 (11.9–14.0)	12.9 (12.6–13.9)	12.3 (11.7–14.0)
**MET—Stage 3**			
20 Hip Hinges + COWAT (completion time [seconds])	36.3 (31.2–41.0)	36.3 (32.4–43.2)	37.2 (28.5–40.5)
COWAT − Hip Hinges (number of words)	7 (5–10)	7 (5–10)	8 (6–8)
20 Lunges + COWAT (completion time [seconds])	39.1 (35.9–41.7)	39.0 (36.9–42.5)	39.1 (33.8–40.1)
COWAT − Lunges (number of words)	7 (5–9)	9 (6–9)	7 (5–7)
20 Squats + COWAT (completion time [seconds])	34.2 (29.3–37.5)	34.7 (33.7–36.9)	29.7 (28.9–36.0)
COWAT − Squats (number of words)	9 (6–11)	9 (6–10)	8 (6–11)
**MET—Stage 4**			
Step Down + Lateral Jump (number of errors /12)	0 (0–0)	0 (0–0)	0 (0–0)
Jump-Overs 1 (number of jump-overs in 20 seconds)	12 (10–15)	11 (10–14)	14 (11–16)
Jump-Overs 2 (number of jump-overs in 20 seconds)	13 (10–14)	12 (10–14)	14 (12–14)
Delayed HVLT (/12)	10 (8–11)	10 (9–10)	10 (8–11)

Data presented as Median (IQR). MET, Multimodal Exertional Test; HVLT, Hopkins Verbal Learning Test; COWAT, Controlled Oral Word Association Test.

### Heart rate

**Figure 2:**
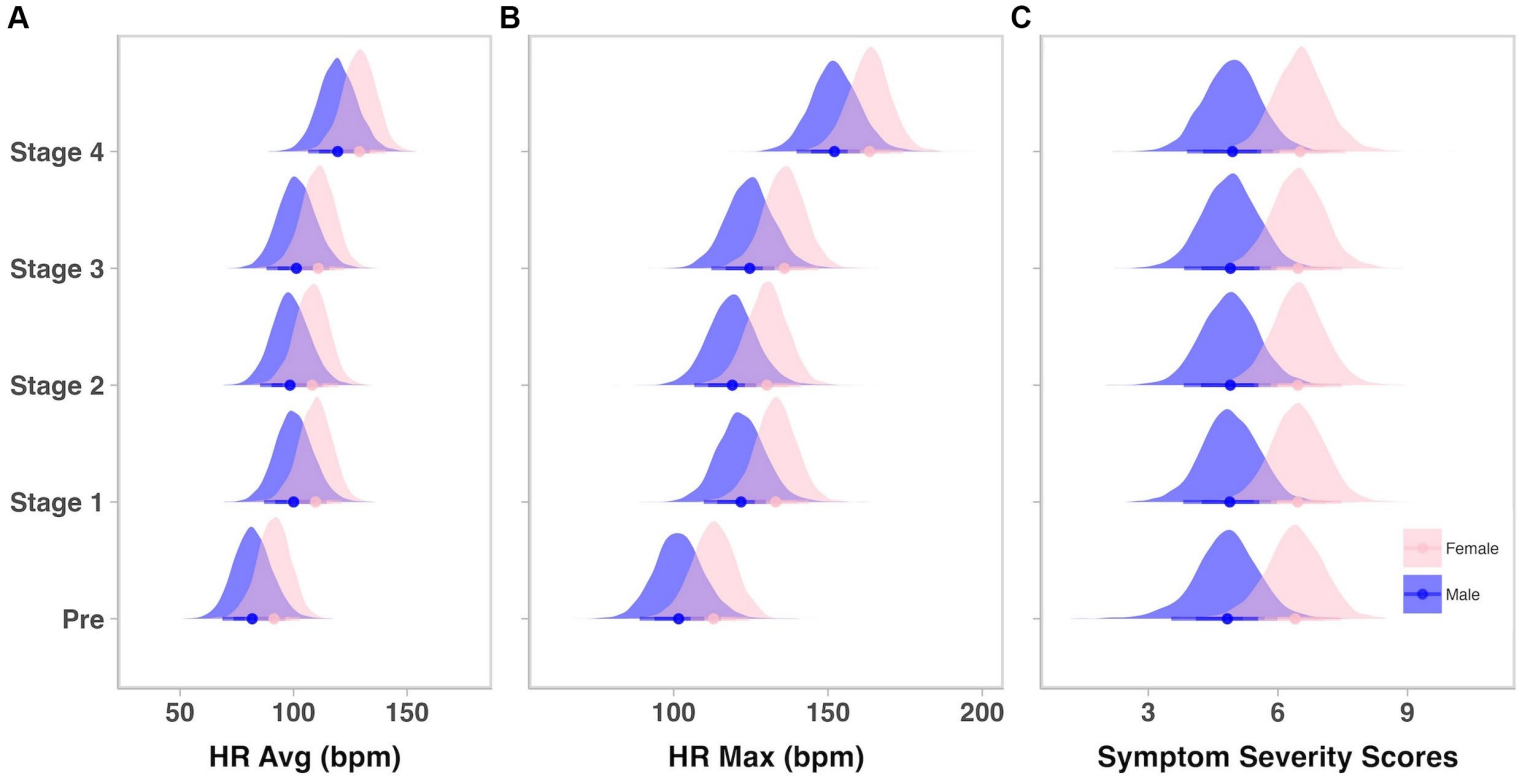
Heart rates, but not symptoms, increase across the four stages of the MET. HR, heart rate; Avg, average; bpm, beats per minute; Max, maximum. Density plots displaying posterior distributions of **(A)** average heart rate, **(B)** maximum heart rate, and **(C)** symptom severity scores between males (blue) and females (pink) prior to (Pre) and during each of the four Multimodal Exertional Test stages. Plots were created from 6,000 posterior draws.

Student-t modeling of HR data showed that the MET elicited an increase in both average HR and maximum HR compared to participants’ pre-test values (Figures 2A,B). The first stage elicited an estimated 18.3 beats per minute (bpm) increase in average HR in all participants (90% CI = 15.6–20.6, posterior probability [pprob] > 0 = 100%). There were no meaningful changes in average HR in Stages 2 and 3, while Stage 4 elicited an estimated increase of 18.1 bpm compared to Stage 3 in all participants (90% CI = 15.7–20.4 bpm, pprob = 100%). Similarly, increases in maximum HR were also seen from pre-MET to Stage 1 (est. average increase = 20.2 bpm, 90% CI = 14.8–25.8 bpm, pprob = 100%) and again from Stage 3 to Stage 4 (est. average increase = 27.6 bpm, 90% CI = 22.6–32.3 bpm, pprob = 100%). Average HR in females was an estimated 9.1 bpm higher compared to males (90% CI = −7.7–25.5 bpm, pprob = 82.2%), and maximum HR was an estimated 11 bpm higher (90% CI = −4.7–25.6 bpm, pprob = 87.7%). Raw values for average HR and maximum HR, before and across the four stages of the MET can be seen in Supplementary Tables 1, 2. With the use of the raw maximum HR values, percentages of age-predicted maximum HRs were calculated using the formula of 220—age (33) and can be found in Supplementary Table 3. Briefly, throughout the four stages of the MET, between 55% and 90% of age-predicted maximum HR was achieved. For a table of estimated average and maximum HR in males and females, please see Supplementary Table 4, and for estimated differences across stages, please see Supplementary Table 5.

### Symptom evaluation

Prior to beginning the MET, Student-t modeling revealed that females initially reported slightly higher symptoms compared to males (avg. = ~6.3 vs. ~4.8, respectively in females and males; avg. diff = 1.5, 90% CI = 0.1–3, pprob = 93.2%; Supplementary Table 4). Altogether, there were very minimal changes in symptom severity in participants across stages or tasks (Figure 2C; Supplementary Table 5). Raw symptom severity scores for males and females at each stage and task can be found in Supplementary Table 6.

## Discussion

The Multimodal Exertional Test (MET) was successfully piloted on healthy interuniversity athletes, demonstrating its ability to incrementally increase and maintain increased HRs across stages without provoking symptoms. Notably, HR increases were observed from pre-MET to Stage 1, and then between Stages 3 to 4, aligning with the expected physical demands of these stages. Female participants exhibited higher HR throughout, indicating a potential sex difference in cardiovascular response during the MET protocol. Minimal symptom provocation supports the protocol’s safety and provides a normative benchmark for symptom levels in healthy, uninjured athletes.

We monitored average and maximum HR across different stages of the MET protocol. Initial observations indicated that Stages 1 and 4 notably increased HR among participants, likely reflecting a progression from rest to activity in Stage 1, and then an intensification of cardiovascular exertion during Stage 4 through plyometric exercises. The consistent heart rates observed across Stages 1 to 3 were not surprising, as these stages aimed to increase head movement and cognitive load rather than cardiovascular demand.

We observed sex-specific differences in HR response: females exhibited higher and more varied HR than males. These preliminary observations underscore the inherent differences in cardiovascular physiology between sexes (34), and one explanation for these differences may be due to the impact of hormonal variations between sexes, specifically from menstrual cycle or contraceptive use (35, 36). Although the limited sample size precluded a thorough exploration of sex-based disparities, these results provide an intriguing direction for future field testing of the MET protocol.

Understanding how the MET impacts symptom burden in healthy athletes is crucial for differentiating between concussion-related symptoms and those arising from normal physical exertion. Our findings indicate minimal symptom provocation during the MET across all participants, with modelled average symptom severity score estimates prior to the MET and throughout all tasks and stages of approximately 5 for males and 6 for females. These estimates are aligned with prior work in healthy athletes (37), as it is common for individuals to report some symptoms owing to fluctuations in daily stress and well-being. The lack of variation in symptom burden across the MET led to expected degeneracies in our statistical modelling; a larger sample and greater detail of participant information will likely overcome this issue and improve future estimates. However, the consistency of symptom scores holds significant clinical relevance, as it will assist us in identifying clinical cut points for test stoppage and can assist healthcare professionals in both clinical decision-making and the future management of individuals with concussions. More specifically, the minimal symptom provocation in healthy athletes provides normative values that can be used for comparison with the responses of individuals with concussion. This is particularly relevant because symptom provocation may signal incomplete recovery and lack of readiness for individuals to return to sport.

### Future directions

Upon completion of the pilot testing and following De Vet et al. (17) six-step framework for the development of a measurement instrument, we reviewed each stage and corresponding tasks to ensure they were aligned with our aim for the MET. The majority of the tasks achieved our desired goals for the stage, however to amplify the cognitive demands of Stage 4’s initial task and to further standardize test administration, we have updated the protocol. Instead of a simple step down followed by a lateral jump based on the examiner’s hand gesture, we have now introduced a cognitive decision-making component. Participants will now perform a lateral jump to the left or to the right contingent on the color of the card presented to them—red for right, blue for left—thereby elevating the task’s cognitive challenge while also standardizing the administration process. Overall, encouraged by the preliminary findings of our pilot work, we embarked on field testing in August 2022. This is the sixth and final step of de Vet et al. (17) framework for measurement tool development and this ongoing process will determine the MET’s re-test reliability, validity, and prognostic capability. We envision the MET protocol as an aid for healthcare professionals in monitoring recovery progression and determining an athlete’s readiness for returning to sport (i.e., unrestricted game play). The results from the present study have provided the initial groundwork for now pursuing field testing with both healthy athletes and those in post-concussion recovery. Future research stemming from these efforts will illuminate the most strategic moments to employ the MET protocol and its potential added value in clinical settings. Additionally, while the statistical models we derived may be overly complicated for the simple design of the current study, their creation was an important and deliberate step not only for deriving estimates in the pilot phase, but to lay the groundwork for normative data mapping and evaluation of future MET studies where we will be estimating responses within a concussion population throughout clinical recovery and beyond.

## Conclusion

The MET has shown promise in its pilot application with healthy athletes, effectively demonstrating the capacity to induce cardiovascular exertion without significant symptom provocation. The MET emerges as an innovative tool that is widely accessible and cost-effective, and may enhance concussion management by integrating cardiovascular stress, cognitive challenges, and coordination tasks with multi-plane movements. This provides a foundation for further validation of the MET and underscores the MET’s potential as a multifaceted measurement instrument in the context of sport-related concussion management.

## Data availability statement

The original contributions presented in the study are included in the article/Supplementary material, further inquiries can be directed to the corresponding author.

## Ethics statement

The studies involving humans were approved by Health Sciences Research Ethics Board, University of Toronto (protocol #41884). The studies were conducted in accordance with the local legislation and institutional requirements. The participants provided their written informed consent to participate in this study.

## Author contributions

KP: Conceptualization, Data curation, Formal analysis, Investigation, Methodology, Project administration, Visualization, Writing – original draft, Writing – review & editing. ADB: Data curation, Formal analysis, Visualization, Writing – original draft, Writing – review & editing. DR: Conceptualization, Writing – original draft, Writing – review & editing. NR: Writing – original draft, Writing – review & editing. DL: Writing – original draft, Writing – review & editing. MH: Conceptualization, Data curation, Funding acquisition, Investigation, Methodology, Project administration, Resources, Supervision, Writing – original draft, Writing – review & editing.

## Footnotes

^1^https://github.com/kylap/Pyndiura_et_al_2024_MET_FNeur.git

## Supplementary material

SUPPLEMENTARY FIGURE 1 Posterior Predictive Check of Student-t model estimating average heart rate. HR, heart rate; Avg, average; bpm, beats per minute. Dot plots facilitating a comparison between the modelled individual average HR predictions (open circles) and the sample data (coloured circles) in males (left panel) and females (right panel) across each stage. Dotted lines represent the estimated group HR averages for males (left panel) and females (right panel). Plots are comprised from 6,000 posterior draws.

Supplementary FIGURE 2 Posterior Predictive Check of Student-t model estimating maximum heart rate. HR, heart rate; Max, maximum; bpm, beats per minute. Dot plots facilitating a comparison between the modelled individual maximum HR predictions (open circles) and the sample data (coloured circles) in males (left panel) and females (right panel) across each stage. Dotted lines represent the estimated group maximum HR for males (left panel) and females (right panel). Plots are comprised from 6,000 posterior draws.

Supplementary FIGURE 3 Posterior Predictive Check of Student-t model estimating symptom severity. Dot plots facilitating a comparison between the modelled individual symptom severity predictions (open circles) and the sample data (coloured circles) in males (left panel) and females (right panel) across each stage and task. Multiple dots with the same colour reflect that each stage is comprised of more than one task. Dotted lines represent the estimated group symptom severity for males (left panel) and females (right panel). Plots are comprised from 6,000 posterior draws.

## References

[1] Patricios JS Schneider KJ Dvorak J Ahmed OH Blauwet C Cantu RC. Consensus statement on concussion in sport: the 6th international conference on concussion in sport–Amsterdam, October 2022. Br J Sports Med. (2023) 57:695–711. doi: 10.1136/bjsports-2023-106898, PMID: 37316210

[2] McCrory P Meeuwisse W Dvorak J Aubry M Bailes J Broglio S. Consensus statement on concussion in sport-the 5th international conference on concussion in sport held in Berlin, October 2016. Br J Sports Med. (2017) 51:838–47. doi: 10.1136/bjsports-2017-097699, PMID: 28446457

[3] McCrory P Meeuwisse WH Aubry M Cantu RC Dvorak J Echemendia RJ. Consensus statement on concussion in sport: the 4th international conference on concussion in sport, Zurich, November 2012. J Athl Train. (2013) 48:554–75. doi: 10.4085/1062-6050-48.4.05, PMID: 23855364 PMC3715021

[4] McCrory P Meeuwisse W Johnston K Dvorak J Aubry M Molloy M. Consensus statement on concussion in sport: the 3rd international conference on concussion in sport held in Zurich, November 2008. J Athl Train. (2009) 44:434–48. doi: 10.4085/1062-6050-44.4.434, PMID: 19593427 PMC2707064

[5] McCrory P Johnston K Meeuwisse W Aubry M Cantu R Dvorak J. Summary and agreement statement of the 2nd international conference on concussion in sport, Prague 2004. Br J Sports Med. (2005) 39:i78–86. doi: 10.1136/bjsm.2005.018614 PMC1725173 15793085

[6] Aubry M Cantu R Dvorak J Graf-Baumann T Johnston K Kelly J. Summary and agreement statement of the first international conference on concussion in sport, Vienna 2001. Br J Sports Med. (2002) 36:6–7. doi: 10.1136/bjsm.36.1.6, PMID: 11867482 PMC1724447

[7] Patricios J, Schneider GM, Ierssel J Purcell LK Davis GA Echemendia RJ. Sport concussion office assessment tool 6 (SCOAT6). Br J Sports Med (2023);57, 651–667, doi: 10.1136/bjsports-2023-106859 37316200

[8] Patricios JS Davis GA Ahmed OH Blauwet C Schneider GM Purcell LK. Introducing the sport concussion office assessment tool 6 (SCOAT6). Br J Sports Med. (2023) 57:648–50. doi: 10.1136/bjsports-2023-106860, PMID: 37316211

[9] Howell DR Buckley TA Lynall RC Meehan WP. Worsening dual-task gait costs after concussion and their association with subsequent sport-related injury. J Neurotrauma. (2018) 35:1630–6. doi: 10.1089/neu.2017.5570, PMID: 29490564

[10] Howell DR Osternig LR Chou LS. Single-task and dual-task tandem gait test performance after concussion. J Sci Med Sport. (2017) 20:622–6. doi: 10.1016/j.jsams.2016.11.020 28169147

[11] Kleiner M Wong L Dubé A Wnuk K Hunter SW Graham LJ. Dual-task assessment protocols in concussion assessment: a systematic literature review. J Orthop Sports Phys Ther. (2018) 48:87–103. doi: 10.2519/jospt.2018.7432, PMID: 29113571

[12] Mitchell CJ Cronin J. Methodological critique of concussive and non-concussive dual task walking assessments: a scoping review. Int J Environ Res Public Health. (2023) 20:227. doi: 10.3390/ijerph20065227, PMID: 36982135 PMC10048786

[13] Marshall CM Chan N Tran P DeMatteo C. The use of an intensive physical exertion test as a final return to play measure in concussed athletes: a prospective cohort. Phys Sportsmed. (2019) 47:158–66. doi: 10.1080/00913847.2018.1542258 30372657

[14] Kochick V Sinnott AM Eagle SR Bricker IR Collins MW Mucha A. The dynamic exertion test for sport-related concussion: a comparison of athletes at return-to-play and healthy controls. Int J Sports Physiol Perform. (2022) 17:834–43. doi: 10.1123/ijspp.2021-0258 35213824

[15] Benedict RHB Schretlen D Groninger L Brandt J. Hopkins verbal learning test—revised: normative data and analysis of inter-form and test–retest reliability. Clin Neuropsychol. (1998) 12:43–55. doi: 10.1076/clin.12.1.43.1726

[16] Brandt J. The Hopkins verbal learning test: development of a new memory test with six equivalent forms. Clin Neuropsychol. (1991) 5:125–42. doi: 10.1080/13854049108403297

[17] de Vet HCW Terwee CB Mokkink LB Knol DL. Measurement in medicine: A practical guide. Cambridge: Cambridge University Press (2011).

[18] Echemendia RJ Meeuwisse W McCrory P Davis GA Putukian M Leddy J. The sport concussion assessment tool 5th edition (SCAT5): background and rationale. Br J Sports Med. (2017) 51:848–50. doi: 10.1136/bjsports-2017-097506, PMID: 28446453

[19] NeuroLogix Technologies. C3Logix: Comprehensive concussion care. (2014). Available at: http://www.c3logix.com/

[20] Bernstein JPK Calamia M Pratt J Mullenix S. Assessing the effects of concussion using the C3Logix test battery: an exploratory study. Appl Neuropsychol Adult. (2019) 26:275–82. doi: 10.1080/23279095.2017.1416471, PMID: 29308917

[21] Stan Development Team. Stan Modelling Language. (2023).

[22] Stan Development Team. RStan: the R interface to Stan. (2023).

[23] R Development Core Team. R: A language and environment for statistical computing. (2023).

[24] RStudio Team. RStudio: Integrated Development Environment for R. (2023).

[25] McElreath R. Rethinking: Statistical Rethinking book package. (2020).

[26] McElreath R. Statistical rethinking: A Bayesian course with examples in R and Stan. 2nd Edn. Chapman and Hall/CRC; (2020) Available at: https://www.taylorfrancis.com/books/9780429642319 (Accessed 5 December 2023).

[27] Vehtari A Gabry J Magnusson M Yao Y Bürkner P Paananen P. loo: Efficient leave-one-out cross-validation and WAIC for Bayesian models. (2023). Available at: https://mc-stan.org/loo/

[28] Wickham H. ggplot2: Elegant Graphics for Data Analysis. (2016).

[29] Gabry J Mahr T. bayesplot: Plotting for Bayesian Models. (2022). Available at: https://mc-stan.org/bayesplot/

[30] Kay M. tidybayes: Tidy Data and Geoms for Bayesian Models. (2021).

[31] Iannone R Cheng J Schloerke B Hughes E Lauer A Seo J. gt: Easily Create Presentation-Ready Display Tables. (2021).

[32] Sjoberg DD Larmarange J Curry M Lavery J Whiting K Zabor EC. gtsummary: Presentation-Ready Data Summary and Analytic Result Tables. (2021).

[33] Fox SM 3rd, Naughton JP. Physical activity and the prevention of coronary heart disease. Prev Med. (1972):1242–50. doi: 10.1016/0091-7435(72)90079-5 PMID: 5069016

[34] St. Pierre SR Peirlinck M Kuhl E. Sex matters: a comprehensive comparison of female and male hearts. Front Physiol. (2022) 13:831179. doi: 10.3389/fphys.2022.831179, PMID: 35392369 PMC8980481

[35] Sims ST Ware L Capodilupo ER. Patterns of endogenous and exogenous ovarian hormone modulation on recovery metrics across the menstrual cycle. BMJ Open Sport Exerc Med. (2021) 7:e001047. doi: 10.1136/bmjsem-2021-001047, PMID: 34367655 PMC8291316

[36] McKinley PS King AR Shapiro PA Slavov I Fang Y Chen IS. The impact of menstrual cycle phase on cardiac autonomic regulation. Psychophysiology. (2009) 46:904–11. doi: 10.1111/j.1469-8986.2009.00811.x, PMID: 19386049 PMC4451597

[37] Hutchison MG Di Battista AP Pyndiura KL Corallo DN Lawrence DW Richards D. Ten-word list performance in healthy athletes and athletes at 3-to-5 days following concussion. Clin J Sport Med. (2021) 32:e354–60. doi: 10.1097/JSM.0000000000000941 34029213

