# Supplementary material for: A Multimodal Exertional Test for concussion: a pilot study in healthy athletes
Source: Front Neurol. 2024 Apr 18;15:1390016. doi: 10.3389/fneur.2024.1390016 (PMC11063232; doi:10.3389/fneur.2024.1390016)
Supplement: Supplementary file 1 [file Data_Sheet_1.zip › Supplementary Methods 1.docx]

***Supplementary Methods 1***

The Multimodal Exertional Test (MET) was developed using De Vet and colleagues'^17^ six-step framework for developing a measurement instrument; in the current study we incorporated the first five steps of this method.

Step 1 – Definition of constructs to be measured:

The MET serves to aid clinicians in determining clinical recovery, prognosticating outcomes, and identifying therapeutic interventions. A literature review was conducted to guide our choice of measurement constructs, which were defined as 1) a subjective component (i.e. symptom reporting), 2) a physiological component (i,e. heart rate), and 3) performance metrics. Our target population was athletic individuals over the age of 13.

Step 2 – Choice of measurement methods:

The MET progressively increases in difficulty at each stage, challenging individuals with more demanding components, including: Stage 1) cardiovascular load, Stage 2) head acceleration, Stage 3) cognitive tasks (i.e., dual-tasks), and Stage 4) elements of coordination and multi-plane movements.

For symptom burden we selected a 27-item Post-Concussion Symptom Scale to evaluate participants’ response of the test in which each symptom would be ranked on a 7-point Likert scale from none to severe (0 indicated ‘none’, 1-2 ‘mild’, 3-4 ‘moderate’, and 5-6 ‘severe’). This symptom evaluation comprises of the 22-item SCAT-5^18^ symptom evaluation with an additional five symptoms including ‘sleeping more than usual’, ‘sleeping less than usual’, ‘difficulty sleeping soundly’, ‘ringing in the ears’, and ‘numbness and tingling’^19,20^. The overall symptom severity is calculated by summing all rated symptoms with a maximum score of 162. To obtain objective, physiological data, we chose to incorporate a heart rate monitor, specifically the Firstbeat chest strap heart rate monitor (Firstbeat Technologies Oy, Jyvaskyla, Finland), to measure heart rate throughout our test. Lastly, for performance metrics, we selected time to completion, total number completed, and/or number of errors as the three metrics that would be used throughout our test.

Step 3 – Selecting items:

We chose three exercises of squats, alternating reverse lunges, and hip hinges to include in the first three stages of our test. The stages differ by the number of repetitions, the addition of a time constraint, or the addition of a cognitive task. For our fourth stage, three tasks were selected that incorporated elements of coordination, agility, decision making, and multi-plane movements, including step downs (from a 10” box) with a lateral jump in the direction of the examiner’s arm, 180º squat jumps, and 180º squat jumps with a go-no-go task.

Step 4 – Scoring items:

Following each MET task, individuals are asked if there were any changes to their symptoms, and a total symptom severity score is calculated for each task. Average and maximum heart rate are calculated before beginning the MET (i.e., pre) and during each of the four MET stages to examine the differences in heart rate between stages. Performance metrics include time to completion, total number of completed, and/or number of errors during each task.

Step 5 – Pilot testing:

The fifth step of pilot testing was split into two parts consisting of feasibility testing and pilot testing. Regarding the former, we completed five rounds of testing to evaluate each of the 12 MET tasks with a new healthy individual. During feasibility testing, we adapted the number of repetitions, altered the last two tasks of our fourth stage to box jump-overs (6” box) with 180º rotation and box jump-overs (6” box) with 180º rotation and a cognitive task. The final version of the MET protocol is displayed in **Figure 1**.
