# Supplementary material for: A Multimodal Exertional Test for concussion: a pilot study in healthy athletes
Source: Front Neurol. 2024 Apr 18;15:1390016. doi: 10.3389/fneur.2024.1390016 (PMC11063232; doi:10.3389/fneur.2024.1390016)
Supplement: Supplementary file 1 [file Data_Sheet_1.zip › Supplementary Methods 2.docx]

***Supplementary Methods 2***

1. y ~ StudentT(ν, 𝜇, σ)

𝜇 = α_stage_ + β_sex_ + γ_participant id_

α ~ Normal(𝜇_α, σ_α)

γ ~ Normal(𝜇_γ, σ_γ)

β ~ Normal(0, 0.4)

𝜇_α, ~ Normal(0, 0.5)

𝜇_γ ~ Normal(0, 0.2)

σ_α , σ_γ ~ Exponential(1)

σ ~ Exponential(1)

ν ~ gamma(2, 0.1)

1. Mathematical Notation for Student-t model to estimate average heart rate or maximum heart rate (y). Stage (α) and participant ID (γ) variables were partially pooled and estimated using a non-centered parameterization.

(2) y ~ StudentT(ν, 𝜇, σ)

𝜇 = α_stage_ + β_sex_ + γ_task_

α ~ Normal(𝜇_α, σ_α)

γ ~ Normal(𝜇_γ, σ_γ)

β ~ Normal(0, 0.4)

𝜇_α, ~ Normal(0, 0.5)

𝜇_γ ~ Normal(0, 0.2)

σ_α , σ_γ ~ Exponential(1)

σ ~ Exponential(1)

ν ~ gamma(2, 0.1)

1. Mathematical Notation for Student-t model to estimate average symptom severity (y). Stage (α) and task (γ) variables were partially pooled and estimated using a non-centered parameterization. Because tasks are specific to the stage in which they are performed, they were explicitly modelled using a nested hierarchical structure. Please see the accompanying STAN code file with the model formulation (<https://github.com/kylap/Pyndiura_et_al_2024_MET_FNeur.git>).

Prior to modelling, all data were transformed to a modified z-score using the median and median absolute deviation. Posterior estimates for average heart rate, maximum heart rate (1) and symptom severity (2) were derived from four Hamiltonian Monte Carlo chains at 3000 iterations per chain. The accompanying model code (<https://github.com/kylap/Pyndiura_et_al_2024_MET_FNeur.git>) also contains prior simulations for each model, which were used to identify regularizing priors that would allow posterior predictions that span the range of scientifically plausible outcomes^25^. Posterior predictive checks for each model were conducted at the participant level to evaluate whether the chosen model for each outcome reasonably approximated the sample (**Supplementary Figures 1-3**). While the models for this manuscript were derived from a simple causal heuristic (see Methods section of main manuscript), a series of additional models were also estimated for comparison to help the reader appreciate how different model formulations (centered vs. non-centered parametrization, partial vs. complete pooling, etc.,) impacted posterior estimates. To aid comparison, models were evaluated for out-of-sample performance and leverage points using Pareto-smoothed importance sampling cross-validation (PSIS) via the “loo” package^27^. Please see the “model_comparison.R” script found at the GitHub repository accompany this manuscript (<https://github.com/kylap/Pyndiura_et_al_2024_MET_FNeur.git>).
