# Supplementary material for: A Multimodal Exertional Test for concussion: a pilot study in healthy athletes
Source: Front Neurol. 2024 Apr 18;15:1390016. doi: 10.3389/fneur.2024.1390016 (PMC11063232; doi:10.3389/fneur.2024.1390016)
Supplement: Supplementary file 1 [file Data_Sheet_1.zip › Supplementary Table 1.docx]

| **Supplementary Table 1. Participants’ raw average heart rates.** | | | |
| --- | --- | --- | --- |
| **Characteristic** | **Overall**, N = 10 | **Female**, N = 6 | **Male**, N = 4 |
| Pre | 81.7 (76.4 – 95.4) | 90.2 (76.4 – 99.7) | 81.7 (79.5 – 82.3) |
| Stage 1 | 100.3 (93.3 – 119.7) | 118.6 (99.0 – 121.9) | 95.2 (93.4 – 98.4) |
| Stage 2 | 98.2 (92.2 – 119.4) | 119.2 (95.6 – 126.0) | 94.4 (92.8 – 97.0) |
| Stage 3 | 101.4 (97.1 – 118.6) | 118.6 (99.1 – 127.9) | 98.3 (97.4 – 100.1) |
| Stage 4 | 119.7 (116.8 – 139.3) | 137.4 (116.1 – 147.3) | 118.5 (117.4 – 119.5) |
| Data presented as Median (IQR). | | | |
