# Supplementary material for: A Multimodal Exertional Test for concussion: a pilot study in healthy athletes
Source: Front Neurol. 2024 Apr 18;15:1390016. doi: 10.3389/fneur.2024.1390016 (PMC11063232; doi:10.3389/fneur.2024.1390016)
Supplement: Supplementary file 1 [file Data_Sheet_1.zip › Supplementary Table 2.docx]

| **Supplementary Table 2. Participants’ raw maximum heart rates.** | | | |
| --- | --- | --- | --- |
| **Characteristic** | **Overall**, N = 10 | **Female**, N = 6 | **Male**, N = 4 |
| Pre | 103.5 (95.8 – 116.8) | 111.5 (97.0 – 122.3) | 101.0 (95.8 – 104.8) |
| Stage 1 | 121.5 (116.5 – 147.3) | 142.5 (117.3 – 153.5) | 119.0 (117.5 – 120.8) |
| Stage 2 | 119.0 (109.3 – 146.8) | 142.5 (115.3 – 151.8) | 113.0 (109.8 – 117.5) |
| Stage 3 | 125.5 (118.3 – 147.3) | 143.5 (119.5 – 157.0) | 123.5 (120.8 – 125.3) |
| Stage 4 | 155.5 (151.0 – 173.0) | 168.0 (152.3 – 177.8) | 151.5 (151.0 – 154.3) |
| Data presented as Median (IQR). | | | |
