# Supplementary material for: A Multimodal Exertional Test for concussion: a pilot study in healthy athletes
Source: Front Neurol. 2024 Apr 18;15:1390016. doi: 10.3389/fneur.2024.1390016 (PMC11063232; doi:10.3389/fneur.2024.1390016)
Supplement: Supplementary file 1 [file Data_Sheet_1.zip › Supplementary Table 3.docx]

| **Supplementary Table 3. Participants’ percentages of age-predicted maximum HR** | | | |
| --- | --- | --- | --- |
| **Stage** | **Formula = 220 – age** | | |
|  | **All** | **Females** | **Males** |
| Stage 1 | 60.8 (58.3 – 73.6) | 71.3 (58.6 – 76.8) | 59.5 (58.8 – 60.4) |
| Stage 2 | 59.5 (54.6 – 73.4) | 71.3 (57.6 – 75.9) | 56.5 (54.9 – 58.8) |
| Stage 3 | 62.8 (59.1 – 73.6) | 71.8 (59.8 – 78.5) | 61.8 (60.4 – 62.6) |
| Stage 4 | 77.8 (75.5 – 86.5) | 84.0 (76.1 – 88.9) | 75.8 (75.5 – 77.1) |
| Data presented as percentages of Median (IQR). | | | |
