# Supplementary material for: A Multimodal Exertional Test for concussion: a pilot study in healthy athletes
Source: Front Neurol. 2024 Apr 18;15:1390016. doi: 10.3389/fneur.2024.1390016 (PMC11063232; doi:10.3389/fneur.2024.1390016)
Supplement: Supplementary file 1 [file Data_Sheet_1.zip › Supplementary Table 4.docx]

| **Supplementary Table 4.** Posterior estimates | | | | | |
| --- | --- | --- | --- | --- | --- |
| **Measure** | **Pre**, N = 6,000 | **Stage 1**, N = 6,000 | **Stage 2**, N = 6,000 | **Stage 3**, N = 6,000 | **Stage 4**, N = 6,000 |
| **Males** | | | | | |
| HR Avg (bpm) | 82.0  (69.0 – 96.5) | 100.3  (86.7 – 114.1) | 98.8  (84.9 – 112.3) | 101.5  (86.7 – 114.1) | 119.6  (106.8 – 134.0) |
| HR Max (bpm) | 101.8  (88.9 – 115.1) | 122.0  (109.5 – 134.5) | 119.1  (106.3 – 131.8) | 124.7  (112.1 – 137.4) | 152.3  (140.0 – 165.5) |
| Symptom Severity | 4.8  (3.6 – 6.0) | 4.9  (3.9 – 6.0) | 4.9  (3.8 – 6.0) | 4.9  (3.9 – 6.0) | 4.9  (3.9 – 6.0) |
| **Females** | | | | | |
| HR Avg (bpm) | 91.2  (78.9 – 103.2) | 109.5  (97.2 – 121.5) | 107.9  (96.2 – 120.6) | 110.7  (99.0 – 123.0) | 128.8  (116.9 – 141.1) |
| HR Max (bpm) | 112.8  (101.3 – 124.7) | 133.0  (122.2 – 144.5) | 130.1  (119.5 – 141.8) | 135.7  (124.4 – 147.0) | 163.3  (152.1 – 174.6) |
| Symptom Severity | 6.3  (5.2 – 7.4) | 6.4  (5.5 – 7.4) | 6.4  (5.5 – 7.4) | 6.4  (5.5 – 7.4) | 6.5  (5.5 – 7.4) |
| Data presented as Mean (90% Compatibility Interval) derived from 6,000 posterior draws. | | | | | |
| HR, Heart Rate; Avg, Average; bpm, beats per minute; Max, Maximum | | | | | |
