# Supplementary material for: A Multimodal Exertional Test for concussion: a pilot study in healthy athletes
Source: Front Neurol. 2024 Apr 18;15:1390016. doi: 10.3389/fneur.2024.1390016 (PMC11063232; doi:10.3389/fneur.2024.1390016)
Supplement: Supplementary file 1 [file Data_Sheet_1.zip › Supplementary Table 5.docx]

| **Supplementary Table 5.** Posterior contrast estimates | | | |
| --- | --- | --- | --- |
| **Measure** | **HR Avg (bpm)**, N = 12,000 | **HR Max (bpm)**, N = 12,000 | **Symptom Severity**, N = 12,000 |
| **Stage 1 - Pre** | 18.3 (15.6 – 20.6) | 20.2 (14.8 – 25.8) | 0.1 (-1.0 – 1.1) |
| **Stage 2 - Stage 1** | -1.6 (-3.9 – 0.6) | -3.0 (-7.6 – 1.6) | 0.0 (-0.9 – 0.9) |
| **Stage 3 - Stage 2** | 2.8 (0.6 – 4.9) | 5.7 (0.5 – 10.1) | 0.0 (-0.9 – 0.9) |
| **Stage 4 - Stage 3** | 18.1 (15.7 – 20.4) | 27.6 (22.6 – 32.3) | 0.0 (-0.9 – 1.0) |
| **Stage 4 - Pre** | 37.6 (34.7 – 40.1) | 50.5 (44.5 – 56.1) | 0.1 (-0.9 – 1.2) |
| Data presented as Mean (90% Compatibility Interval) from 12,000 posterior draws. | | | |
| HR, Heart Rate; Avg, Average; bpm, beats per minute; Max, Maximum | | | |
