# Supplementary material for: A Multimodal Exertional Test for concussion: a pilot study in healthy athletes
Source: Front Neurol. 2024 Apr 18;15:1390016. doi: 10.3389/fneur.2024.1390016 (PMC11063232; doi:10.3389/fneur.2024.1390016)
Supplement: Supplementary file 1 [file Data_Sheet_1.zip › Supplementary Table 6.docx]

| **Supplementary Table 6. Participants’ raw differences from initial symptom severity score.** | | | |
| --- | --- | --- | --- |
| **Characteristic** | **Overall**, N = 14 | **Female**, N = 8 | **Male**, N = 6 |
| **Initial** | | | |
| Symptom Severity Score | 4 (2 – 8) | 6 (4 – 9) | 3 (1 – 5) |
| **MET - Stage 1** | | | |
| 20 Hip Hinges | 0 (0 – 1) | 0 (0 – 0) | 1 (1 – 1) |
| 20 Lunges | 0 (0 – 1) | 0 (0 – 0) | 1 (1 – 1) |
| 20 Squats | 0 (0 – 1) | 0 (0 – 0) | 1 (1 – 1) |
| **MET - Stage 2** | | | |
| 10 Hip Hinges | 0 (0 – 1) | 0 (0 – 0) | 1 (1 – 1) |
| 10 Lunges | 0 (0 – 1) | 0 (0 – 0) | 1 (1 – 1) |
| 10 Squats | 0 (0 – 1) | 0 (0 – 0) | 1 (1 – 1) |
| **MET - Stage 3** | | | |
| 20 Hip Hinges + COWAT | 0 (0 – 1) | 0 (0 – 0) | 1 (1 – 1) |
| 20 Lunges + COWAT | 0 (0 – 1) | 0 (0 – 0) | 1 (1 – 1) |
| 20 Squats + COWAT | 0 (0 – 1) | 0 (0 – 0) | 1 (1 – 1) |
| **MET - Stage 4** | | | |
| Step Down + Lateral Jump | 0 (0 – 1) | 0 (0 – 0) | 1 (1 – 1) |
| Jump-Overs 1 | 0 (0 – 1) | 0 (0 – 0) | 1 (1 – 2) |
| Jump-Overs 2 | 0 (0 – 1) | 0 (0 – 0) | 1 (1 – 2) |
| Data presented as Median (IQR). | | | |
| MET, Multimodal Exertional Test; COWAT, Controlled Oral Word Association Task | | | |
