# Supplementary figures and images for: A Multimodal Exertional Test for concussion: a pilot study in healthy athletes
Source: Front Neurol. 2024 Apr 18;15:1390016. doi: 10.3389/fneur.2024.1390016 (PMC11063232; doi:10.3389/fneur.2024.1390016)

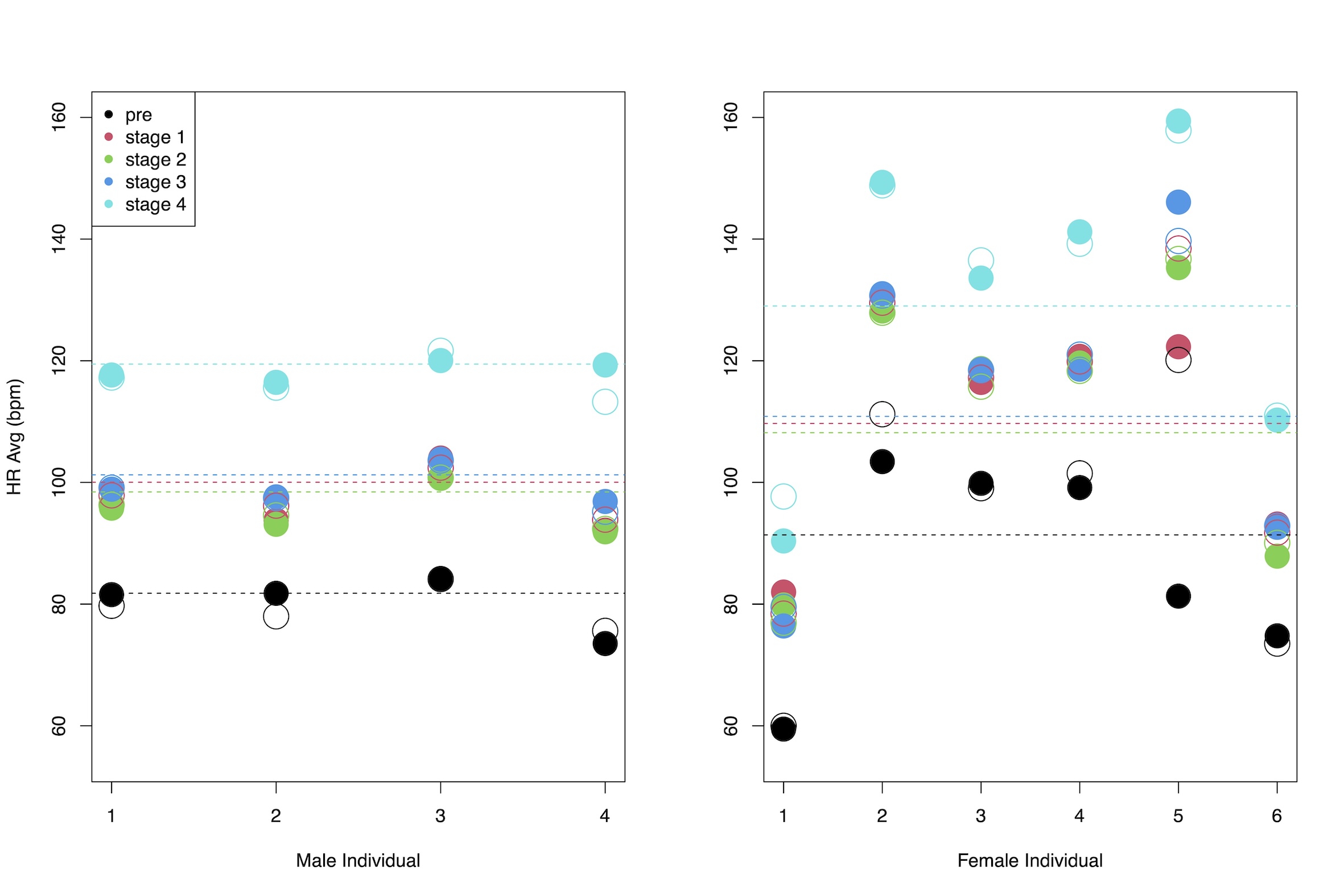

Supplement: Supplementary file 1 [file Data_Sheet_1.zip › Supplementary Figure 1.jpeg]

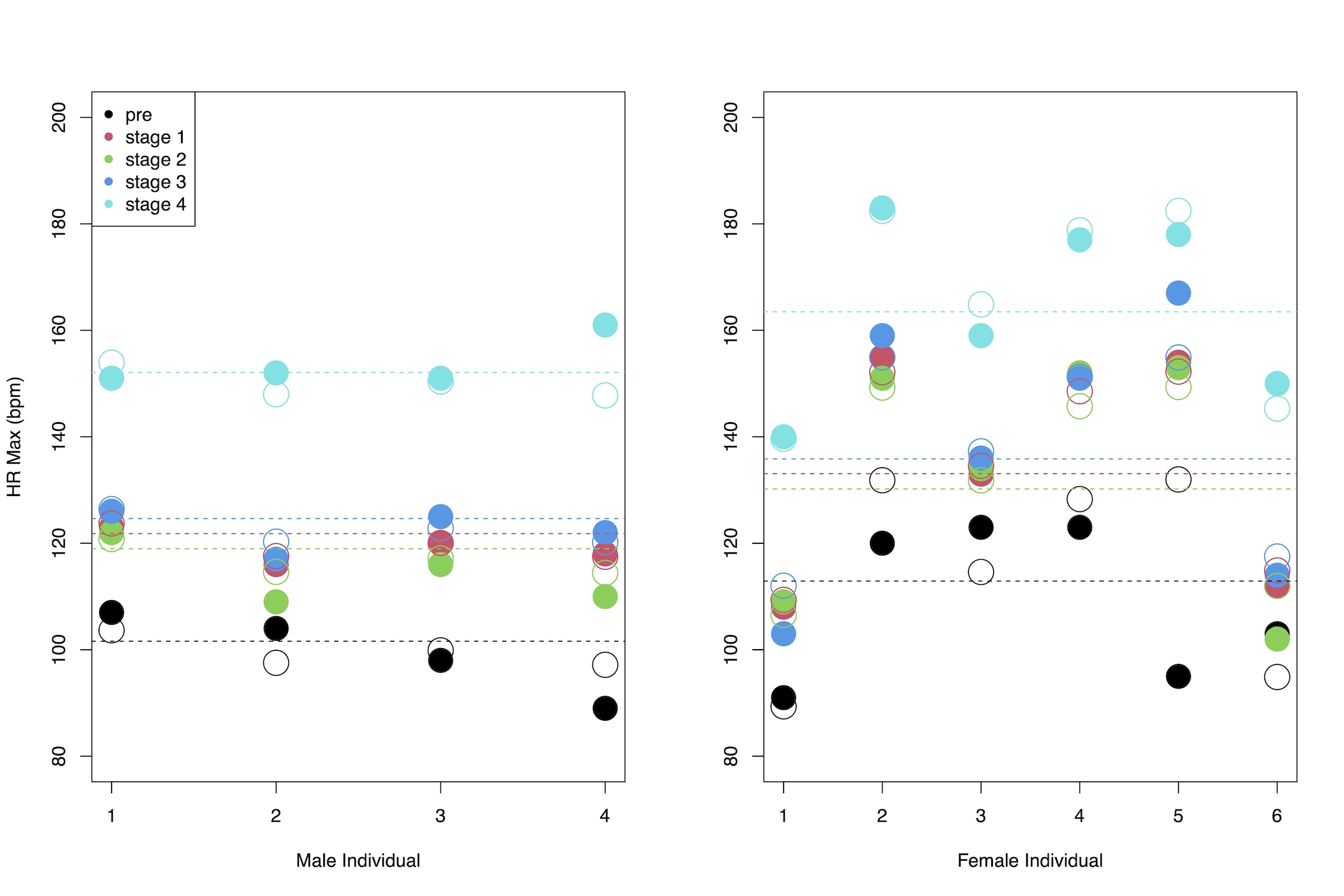

Supplement: Supplementary file 1 [file Data_Sheet_1.zip › Supplementary Figure 2.jpeg]

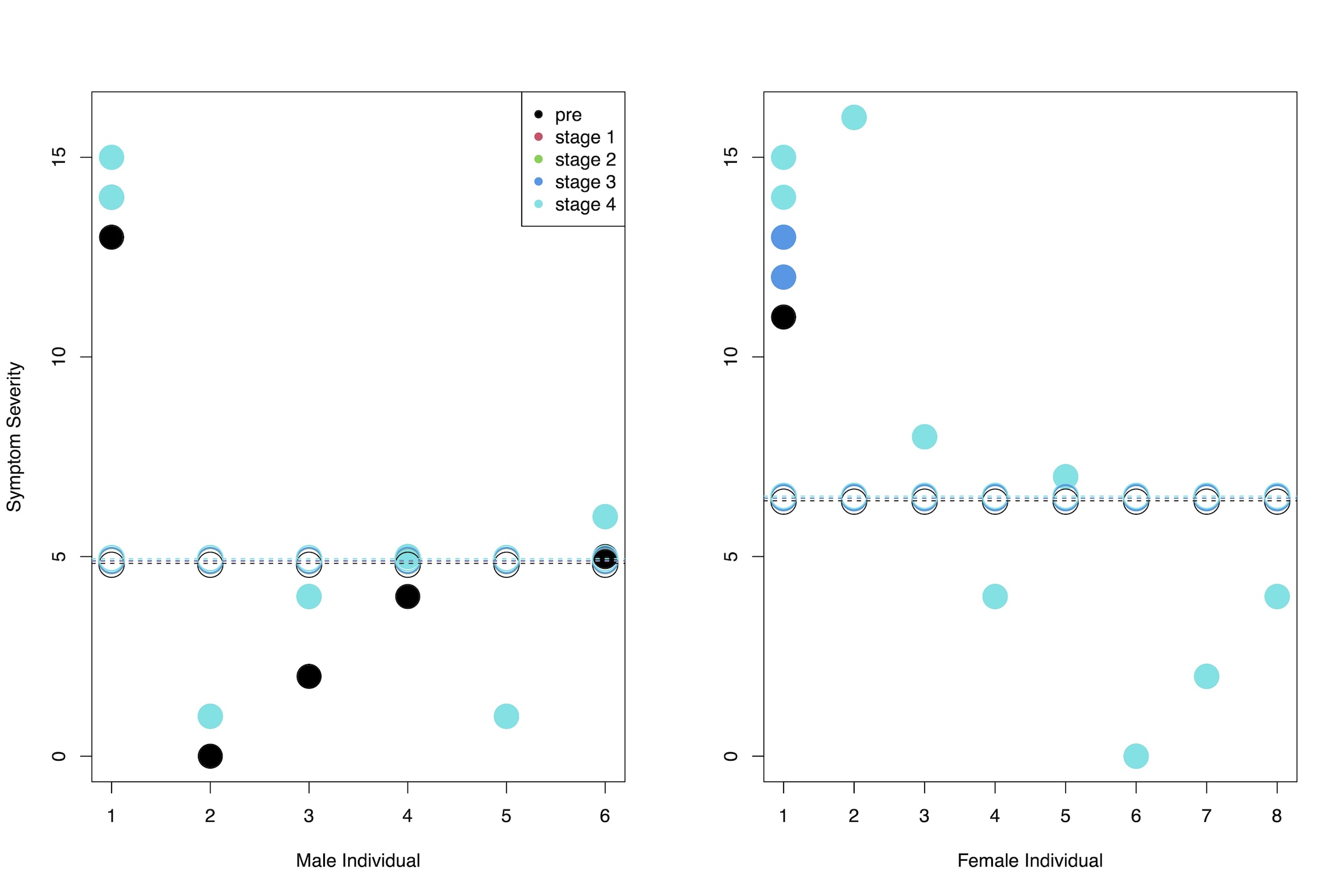

Supplement: Supplementary file 1 [file Data_Sheet_1.zip › Supplementary Figure 3.jpeg]
